# New Hybrid Nanocomposites with Catalytic Properties Obtained by In Situ Preparation of Gold Nanoparticles on Poly (Ionic Liquid)/Poly (4-Vinylpyridine) Nanofibers

**DOI:** 10.3390/polym14183782

**Published:** 2022-09-09

**Authors:** Oscar Ramírez, Matías Leal, Ximena Briones, Marcela Urzúa, Sebastián Bonardd, Cesar Saldías, Angel Leiva

**Affiliations:** 1 Departamento de Físico Química, Facultad de Química y de Farmacia, Pontificia Universidad Católica de Chile, Santiago 7820436, Chile; 2 Centro de Bioinformática y Biología Integrativa, Facultad de Ciencias de la Vida, Universidad Andrés Bello, Santiago 8370035, Chile; 3 Departamento de Química Orgánica y Fisicoquímica, Facultad de Ciencias Químicas y Farmacéuticas, Universidad de Chile, Santiago 8380544, Chile; 4 Departamento de Química, Facultad de Ciencias, Universidad de Chile, Santiago 7800003, Chile; 5 Departamento de Química Orgánica, Universidad de La Laguna, Avda. Astrofísico Francisco Sánchez 3, La Laguna, 38206 Tenerife, Spain; 6 Instituto de Bio-Orgánica Antonio González, Universidad de La Laguna, Avda. Astrofísico Francisco Sánchez 2, La Laguna, 38206 Tenerife, Spain

**Keywords:** poly ionic liquids, nanofibers, electrospinning, gold nanoparticles, hybrid nanocomposites

## Abstract

In this work, we report the obtaining of new hybrid nanocomposites with catalytic activity formed by nanofibers of polymer blends and gold nanoparticles. The nanofibers were obtained by electrospinning blends of a poly (ionic liquid) (PIL) and its precursor polymer, poly (4-vinyl pyridine) (P4VPy). The characteristics of the nanofibers obtained proved to be dependent on the proportion of polymer in the blends. The nanofibers obtained were used to synthesize, in situ, gold nanoparticles on their surface by two-step procedure. Firstly, the adsorption of precursor ions on the nanofibers and then their reduction with sodium borohydride to generate gold nanoparticles. The results indicated a significant improvement in the performance of PIL-containing nanofibers over pure P4VPy NFs during ion adsorption, reaching a 20% increase in the amount of adsorbed ions and a 6-fold increase in the respective adsorption constant. The catalytic performance of the obtained hybrid systems in the reduction reaction of 4-nitrophenol to 4-aminophenol was studied. Higher catalytic conversions were obtained using the hybrid nanofibers containing PIL and gold nanoparticles achieving a maximum conversion rate of 98%. Remarkably, the highest value of kinetic constant was obtained for the nanofibers with the highest PIL content.

## 1. Introduction

The rapid development of nanoscience and nanotechnology in recent decades has led to significant advances in the applications of nanomaterials in many technological fields. Thus, the special physical and chemical properties of nanomaterials have been widely explored to develop or improve applications aimed at solving important current global challenges, such as environmental remediation, sustainable energy, and climate change. Indeed, a great deal of scientific activity has been developed to obtain nanomaterials able to contribute, for example, to capture and efficiently use carbon dioxide from the air, degrading toxic pollutants in water and contributing to process of energy conversion and storage [1,2,3]. Thus, the use of catalytic materials can help to improve the efficiency of diverse processes, their economy, and even contribute to reduce the generation of waste and pollutants. In this context, the exploration and development of advanced materials to be used in nanocatalysis represents an important challenge in the short-medium term, in order to contribute to sustainable chemistry [4].

Thanks to the important achievements of nanoscience and nanotechnology, it is now possible to prepare with great precision many catalytic materials at the nanometer scale, achieving a significant improvement in their performance thanks to the so-called “nanoeffects”. These nanoeffects, although not fully understood to date, are attributed to structural, electronic and quantum size effects [5]. Nanocatalysts can be formed by composites, compounds, alloys, or elemental solids and can also be obtained in different formats (e.g., powder, thin films, fibers). Among the nanomaterials with catalytic properties, nanoparticles (NPs) of noble metals stand out, since, in addition to providing high surface areas favoring the interaction between the reactants and the catalyst surface, they display their unique chemical, electronic, and optical properties through the well-known plasmonic effect [6].

Notwithstanding the above, the use of nanomaterials as catalysts also presents some difficulties. For example, a considerable decrease in the activity of catalytic NPs has been observed after a few cycles of use, which has been attributed to aggregation and surface passivation phenomena. Indeed, when NPs agglomerate, they tend to lose their nanometer size and transform into bulk materials losing their special properties. Another problem really challenging is the difficulty to recover the catalytic nanoparticles from the reaction medium for further use. To solve these problems a suitable strategy is to support the nanoparticles on structures of different type and nature [7], achieving that the supports not only facilitate the recovery and reuse of the nanocatalyst, but also prevent the aggregation of the NPs during successive reaction cycles or under continuous flow conditions. Interestingly, with the advancement of knowledge it has been established that in many cases the role played by the supports in practice may be rather more complex and even not yet clearly understood. Some supports can perform, directly or indirectly, different functions during a catalytic reaction, providing specific defect sites. These zones would allow to anchor and even stabilize metal nanoparticles favoring activity and transfer of electrons [8]. 

In general, it is desirable that the ideal supports are chemically inert, have a large surface area and uniform size, and retain the nanocatalyst efficiently [9]. Among the most used supports for nanocatalyst immobilization are inorganic fibers, glass mats, and different polymers [10]. An alternative that has shown great promise for supporting and stabilizing nanoparticles, preserving their special properties over time and after repeated use in different chemical environments, is the use of polymeric nanofibers (NFs) [11,12]. One of the advantages offered by polymeric NFs is that these materials can be obtained on a macroscopic scale in the form of mat-like structures. These mats are characterized by high flexibility and elasticity, larges specific surface area, and show high degree of porosity, of small pore sizes and high pore interconnectivity. In addition, it has been demonstrated that electrospun nanofiber films can be modified and grafted with different functionalities, providing them with a high potential to be used for various purposes. Thanks to these special properties, polymeric nanofiber mats can be advantageously used in a variety of emerging environmental applications, such as air filtration, heavy metal ion adsorption, self-cleaning applications, and water purification processes. Consequently, considerable effort has been made to obtain hybrid catalytic nanomaterials of the Polymer Nanofibers containing metal Nanoparticles, in which the special characteristics of both nanocatalysts and nanofibers converge [12,13].

Inspired by the above, we have prepared a hybrid nanomaterial consisting of polymeric nanofibers and gold nanoparticles, and we studied its performance as a heterogeneous catalyst using the well-known reduction reaction of 4-nitrophenol (4NP) as a model. Noticeably, in this simple reduction reaction with sodium boron hydride catalyzed by gold nanoparticles, 4-NP, a compound considered a priority pollutant by the USEPA, is effectively reduced to form the considerably less toxic compound 4-aminephenol (4-AP) under mild reaction conditions, and the reaction can be easily monitored by UV-vis spectroscopy [14,15].

The polymeric nanofibers were obtained by electrospinning mixtures of the poly ionic liquid, Poly penthyl 4-vinylpyridine bis trifluoromethane sulfonamide (PP4VPy-NTf_2_^−^) and poly 4-vinyl pyridine PV4Py, while the gold nanoparticles were synthesized in situ on the nanofibers by reduction in ionic precursors adsorbed on them. The polymeric ionic liquids PILs, also known as poly (ionic liquids) are a special type of polyelectrolytes that contain some of the chemical groups of ILs, cations and anions. PILs keep some of the special properties of Ils, such as, negligible vapor pressure, ionic conductivity, thermal stability, tuneable solution properties, nonflammability, and high chemical and electrochemical stabilities, as well as good compatibility. Many of the special properties that characterize ILs are mainly attributable to their ionic nature and are, therefore, also present in PILs. A particularly interesting property of PILs is their high capacity to interact and their compatibility with different materials, both organic and inorganic, hybrids, e.g., metal-organic and biomacromolecules [16,17]. Based on the above, PILs are expected to present a good performance as support for catalytic Au nanoparticles, mainly due to the potential generation of favorable interactions between NFs and NPs, providing high stability to the system along with other special properties such as flexibility, processability and mechanical durability, all highly desirable for supported catalysts. Therefore, the main novelty and also the main aim of this work lies in the inclusion of a PIL in the nanofibers and the study of its effect in the in situ gold nanoparticles and in the catalytic performance of the final hybrid nanomaterial. To the best of our knowledge, to date, information on the use of PIL-containing NFs as supports for catalytic NPs is at least scarce, so we seek to contribute to knowledge in this interesting area.

## 2. Materials and Methods

### 2.1. Materials

Poly (4-vinylpyridine) (P4VPy) ${\bar{M}}_{n}$ *=* 160,000 g mol^−1^, potassium tetrachloroaurate (III) (KAuCl_4_, 99.9%), bis(trifluoromethane)sulfonamide lithium salt (LiNTf_2_, 99.9%) and pentyl bromide (99.9%) were purchased from Sigma−Aldrich. Ethyl acetate, methanol, *N*, *N*-dimethyl formamide (DMF), dichloromethane (DCM) and sodium borohydride (NaBH_4_, 98.0%) were purchased by Merck. Milli-Q water (18.2 MΩ/cm) was used in all required experiments. All solvents used were of analytical grade and used without further purification.

### 2.2. Synthesis of Poly(Ionic Liquid) PP4VPy-NTf_2_^−^

Poly (pentyl 4-vinylpyridine bis trifluoromethane sulphonamide) (PP4VPy-NTf_2_^−^) was synthesized by a two-step process previously reported [18]. Firstly, the quaternization of pyridine rings was carried out by using pentyl bromide as alkylating agent. Then, in a 50 mL round-bottom flask, the counterion exchange was carried out by dissolving 1 g of quaternized PP4VPy-Br^−^ in 20 mL of Milli-Q water and adding a LiNTf_2_ aqueous solution containing 6 g of salt in 12 mL of water. After stirring for 30 min at room temperature, the white precipitated formed was filtered, washed several times with water and finally lyophilized.

### 2.3. Nanofibers Preparation 

Polymer nanofibers were obtained by electrospinning of PP4VPy-NTf_2_^−^ and P4VPy blend solutions in a DMF/DCM (1:1 *v*/*v*) solvent mixture. The solutions were prepared in 100/0, 90/10, 80/20, 70/30, and 50/50 ratios of P4VPy/PP4VPy-NTf_2_^−^, maintaining a total polymer concentration of 20% *w*/*v*, and were stirred at room temperature during 24 h before electrospinning. A Linari Engineering electrospinning system was used, the operating parameters were a voltage of 15 kV, a tip-collector distance between 10–20 cm and a flow rate of 1.5 mL/h.

### 2.4. FTIR and 1H-NMR Analysis 

FTIR analysis of samples was performed using a VECTOR 22 FTIR (Bruker, Germany). FTIR spectra were recorded between 600–4000 cm^−1^ after the accumulation of 20 scans with a resolution of 2 cm^−1^. ^1^H-NMR and ^19^F-NMR measurements were carried out at room temperature using a BRUKER AVANCE III HD-400 spectrometer (400 MHz). Samples were prepared by dissolving 50 mg of material in DMSO-d6 (0.5 mL).

### 2.5. Conductimetric Measurements

The electrical conductivity of polymer solutions was performed using a digital conductivity meter CX-701 (Agrotools meettechniek) with a glass electrode and a cell constant of 1 cm^−1^. The conductivity of the electrode was first calibrated using a KCl solution with a conductivity (σ) of 1.41 mS/cm. 

### 2.6. Thermogravimetric Analysis

TGA measurements were performed in a Mettler TGA/SDTA 851^e^ calorimetric system and processed using the STAR^e^ program interface. Measurements were carried out in alumina pans by heating the sample between 25 and 800 °C at 20 °C/min under a nitrogen flow of 20 mL/min.

### 2.7. Scanning Electron Microscopy FESEM

The visualization of obtained nanofibers was carried out using field emission scanning electron microscopy (FE-SEM) on a FEI QUANTA FEG 250 microscope equipped with an Oxford X-MAX50 energy dispersive X-ray spectroscopy (EDX) analyzer. Nanofibers were collected on silicon wafers and coated with an ultrathin layer of gold. Electron micrographs were recorded in high-vacuum mode under an acceleration voltage of 10.0 kV. The analysis of the morphology, average-diameter size, and diameter size distribution was performed using the Image-J image processing program.

### 2.8. X-ray Photoelectron Spectroscopy

X-ray photoelectron spectroscopy (XPS) spectra were collected by a SPECS equipped with an PHOIBOS 150 analyzer, 1D-DLD detector and a focus 500 monochromatic excitation source (X-ray Al Kα hν = 1486.71 eV) using a Flood gun to compensate charge effects. 

### 2.9. AuCl_4_^−^ Adsorption Experiments on PP4VPy-NTf_2_^−^/P4VPy Nanofibers

Measurements were performed using 10 mL of a KAuCl_4_ 40 ppm stock solution and 10 mg of nanofiber mat. Adsorption kinetics were carried out by using UV-visible spectroscopy, and following the intensity decrease in the band centered at 305 nm, ascribed to the AuCl_4_^−^ specie. Aliquots from the supernatant were analyzed at different time intervals until reaching an equilibrium-state. The gold concentration was determined by UV-visible spectroscopy using a calibration curve. The adsorption capacity, *q_t_* (mg/g) was calculated using the following equation:

(1) $$q_{t}=\left(\frac{C_{0}-C_{t}}{m}\right)V_{0}$$

where *C_0_* and *C_t_* correspond to the initial gold concentration and the concentration measured at time t. *V_0_* is the initial volume of solution and *m* is the mass of adsorbent used in the adsorption.

### 2.10. Synthesis of Gold Nanoparticles Supported on PP4VPy-NTf_2_^−^/P4VPy Nanofibers

The preparation of PP4VPy-NTf_2_^−^/P4VPy nanofibers containing gold nanoparticles was achieved following a two-step consecutive process. Firstly, a nanofiber mat (10 mg) was immersed in 10 mL of a 40 ppm KAuCl_4_ solution and kept with gentle stirring for 1 h. The complete adsorption was confirmed by UV-visible spectroscopy corroborating the disappearance of the band at 305 nm. Once the adsorption was concluded, the nanofiber mat was washed with abundant water for 1 h and dried under vacuum for 24 h at 35 °C. In a second step, the reduction in the adsorbed gold ions was carried out using NaBH_4_ as reducing agent. Nanofiber mats were immersed into 10 mL of Milli-Q water under constant stirring for 10 min. Then, 0.5 mL of a NaBH_4_ solution (25 mg/mL) was added, visualizing immediately a color change in the material, going from pale-yellow to purple, accusing the formation of gold nanoparticles. After 30 min of reaction, nanofibers mats were removed, washed with abundant water, and dried under vacuum for 24 h at 35 °C. 

### 2.11. Catalytic Studies Based on the 4-Nitrophenol (4NP) Reduction

The catalytic activity of nanocomposites was tested using, as a model reaction, the well-known catalyzed reduction in 4NP, using a methodology previously reported by our group [19]. Briefly, 5 mL of a stock solution of 4NP (60 μM) and 1 mg of catalyst were placed into a glass vial equipped with a magnetic stirrer. Subsequently, 350 µL of a 20 mg/mL NaBH_4_ solution was added to start the reaction. The evolution of the reaction was studied by UV-Vis spectroscopy by following the disappearance of the band centered at 400 nm, assigned to the p-nitrophenolate specie. The data were analyzed by adjusting the results to a pseudo-first order kinetics. 

## 3. Results and Discussion

**Figure 1:**
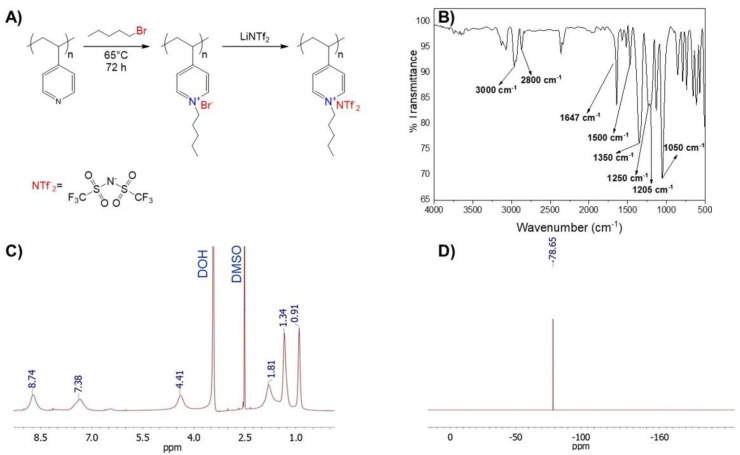
(**A**) Scheme of synthesis PP4VPyNTf_2_^−^ poly(ionic liquid) synthesis; (**B**) FT-IR spectrum and (**C**) ^1^H- and (**D**) ^19^F- NMR spectra of the poly(ionic liquid).

For the poly ionic liquid PP4VPy-NTf_2_^−^ synthesis, initially, P4VPy was reacted with an excess of pentyl bromide (alkylating agent) to achieve almost complete quaternization of the pyridinyl groups. A quaternization degree of 99% was obtained by FTIR and ^1^H-NMR analysis, as reported in a previous work [18]. Then, the exchange of bromide for NTf_2_^−^ anions, was performed by direct dissolution of the LiNTf_2_ salt on a solution of the polyelectrolyte PP4VPy-Br [20]. Finally, purification of PP4VPy-NTf_2_^−^ PIL was performed by consecutive dialysis, filtration and lyophilization. The successful synthesis of PP4VPy-NTf_2_^−^ was corroborated by FTIR, ^1^H-NMR and ^19^F-NMR product characterization. A scheme of synthesis route and the corresponding spectra are shown in Figure 1.

The FTIR spectrum (Figure 1B) shows the typical bands expected for the quaternized PP4VPy^+^ backbone, accompanied by new ones ascribed to vibrational modes of NTf_2_^−^ counterions. The quaternization process was verified by the appearance of an intense band at 1647 cm^−1^ corresponding to the vibrational modes of the quaternized pyridine rings, and the absence of the signal attributed to the vibrations of the uncharged pyridine rings which typically appears at 1600 cm^−1^. Moreover, the increased intensity of bands between 2800–3000 cm^−1^, would denote a higher aliphatic content in the polymer due to the incorporation of pentyl moieties. On the other hand, the absorption bands at 1350, 1250, 1205, 1130, and 1050 cm^−1^, are ascribed to NTf_2_^−^ counterions. Figure 1C shows the ^1^H-NMR spectrum of PP4VPyNTf_2_^−^, the most relevant signals are, at 8.74 and 7.38 ppm corresponding to protons of the quaternized pyridine rings, and signals at 1.81, 1.34, and 0.91 ppm protons of pentyl chains attached to the nitrogen atom of pyridine groups. Notably, the analysis of ^1^H-NMR spectrum indicated a quantitative polymer quaternization. Similarly, the ^19^F-NMR spectrum analysis allowed corroborate the presence of NTf_2_^−^ counterions (specifically, −CF_3_ signal at 78.65 ppm) in the PIL structure. 

**Figure 2:**
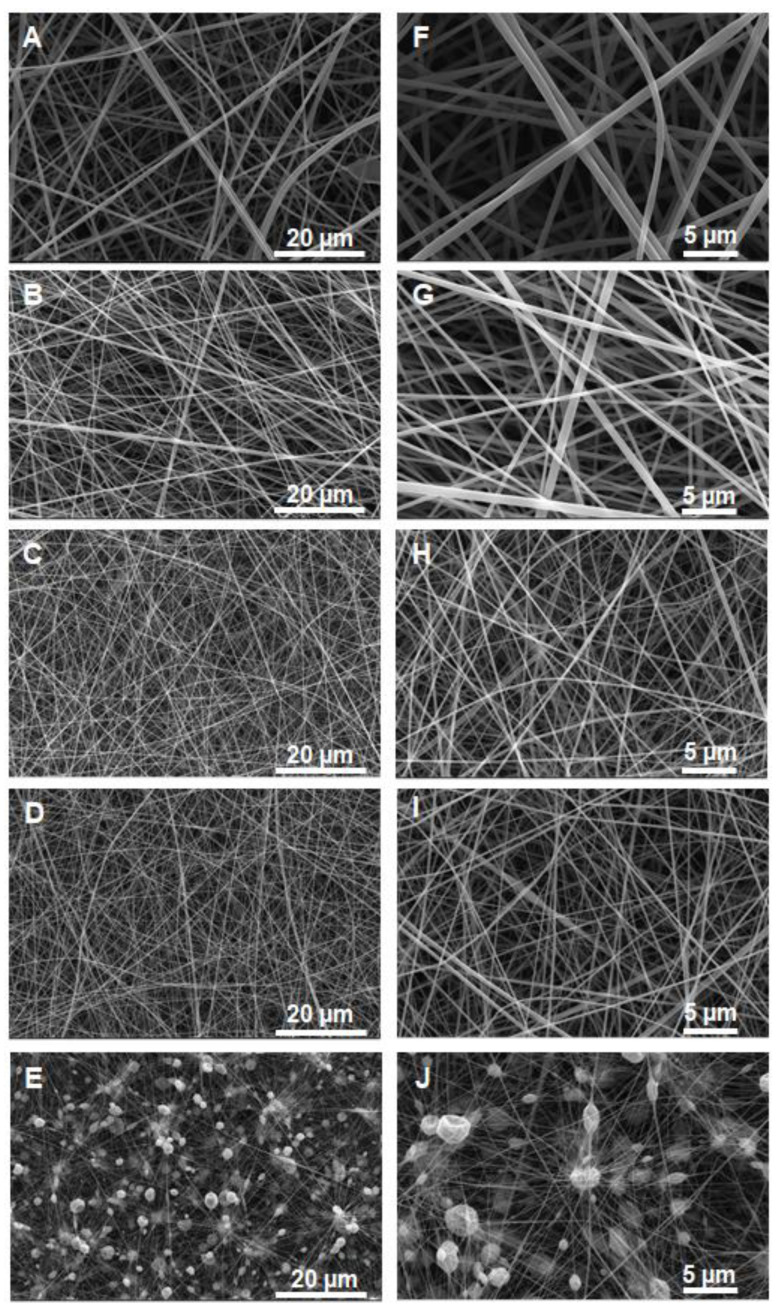
FE-SEM micrographs of pristine P4VPy nanofibers (**A**) and P4Vpy/PP4VpyNTf_2_^−^ nanofibers with PIL compositions of 10 (**B**), 20 (**C**), 30 (**D**), and 50% *w*/*w* (**E**). (**F**–**J**) the same with higher resolution.

Polyelectrolytes are among the most difficult polymers to be electrospun since this type of polymer has special properties in solution due to the presence of charges along their chains [21]. To minimize this difficulty, nanofibers were obtained by electrospinning employing solutions prepared from mixtures of PP4VPy-NTf_2_^−^ and P4VPy in different proportions, using DMF/DCM (1:1 *v*/*v*) as solvent. Figure 2 shows SEM images of the obtained nanofibers obtained from the P4VPy and P4VPy/PP4VPyNTf_2_^−^ blends.

**Figure 3:**
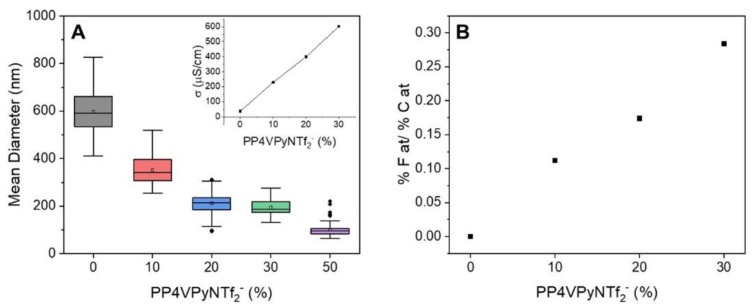
(**A**) Diameters Distribution of P4VPy and P4VPy/PP4VPyNTf_2_^−^ nanofibers and corresponding solution conductivities (insert). (**B**) EDX analysis of the relative content of fluorine respect to carbon atoms, % F at/% C at.

Images revealed the obtainment of uniform and bead-free nanofibers using pure P4VPy and their blends with compositions up to 30% *w*/*w* of PP4VPyNTf_2_^−^. In contrast, the blend containing 50% *w*/*w* of PIL produced a material consisting of a high content of beads connected to each other by rather irregular fibers. A significant detriment of the mechanical properties of fibrous materials accompanied by a drastic effect on their surface properties due to the presence of beads have been reported [22]. Notably, by increasing the amount of PIL in blends, mats with higher fiber density and lower fiber diameter values were obtained, Figure 3A. In addition, the analysis of the size distributions shows that the diameter of the nanofibers becomes narrower as the PIL content in the electrospun solution increases. These results are ascribed to the increase in conductivity of the solutions as the PIL content increases (insert Figure 3A), giving rise to an increase in the repulsive forces in the solution jet during the electrospinning process, phenomenon that significantly reduce the diameter of the fibers [23]. On the other hand, a proper relation between the PIL content of solutions and obtained nanofibers was determined by means of EDX analysis, with a rising trend for the values of relative content of fluorine regarding carbon (% F/% C), as is shown in Figure 3B. 

**Figure 4:**
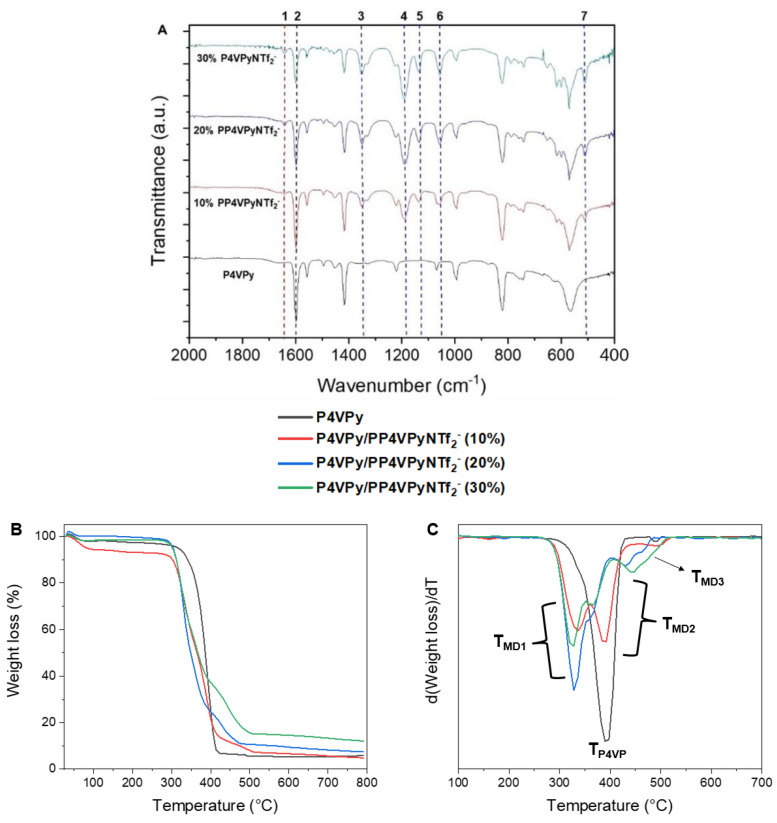
(**A**) FT-IR spectra of the P4VPy and P4VPy/PP4VPyNTf_2_^−^ nanofibers, (**B**) thermal decomposition profiles of P4VPy and P4VPy/PP4VPNTf_2_^−^ nanofibers, and (**C**) curves of the corresponding weight loss derivatives with temperature.

The nanofibers obtained with different amounts of PIL were characterized by FTIR spectroscopy and thermogravimetric analysis (TGA). Figure 4A shows the FTIR spectra of bare P4VPy NFs and of all P4VPy/PP4VPyNTf_2_^−^ blend NFs. The characteristic signal centered at 1600 cm^−1^ (signal 2 in spectra) which correspond to vibrations of the non-quaternized pyridine rings, are observed in all nanofibers. Additionally, new signals ascribed to PIL are detected. As in the PIL spectrum (Figure 1B), the FTIR spectra of the P4VPy/PP4VPyNTf_2_^−^ NFs present a signal attributed to the quaternized pyridine rings, labelled as 1, and signals corresponding to the NTf_2_^−^ counterions, from 3 to 7. As expected, these signals showed an intensity increase in those blends having higher PIL content. 

Figure 4B,C show the thermal degradation profiles and their corresponding differential curves of the nanofibers of P4VPy and P4VPy/PP4VPyNTf_2_^−^ blends. From these results, relevant information such as onset degradation temperatures (T_onset_), maximum decomposition rate temperatures (T_MAX_), and percentage of residues were obtained. The above-mentioned values are summarized in Table 1. 

All samples showed an initial weight loss at around 100 °C, which is attributed to the removal of water molecules. All nanofibers except P4VPy exhibited a multistage degradation profile consisting of three stages. Reported works indicate that the first stage could be related to the degradation of the quaternized pyridinyl moieties since, apparently, the quaternization process induces a selective labilization of the pyridine structure [24]. On the other hand, the second stage would correspond to the decomposition of pentyl chains. Interestingly, the temperatures of this stage in the blends containing 20 and 30% PIL are lower than that of blend containing 10% of PIL. The loss weight of third stage, would be related to the volatilization of fragments coming from NTf_2_^−^ counterions [25]. This is consistent with the intensity increase in DTGA curves as the PIL content increases. Although the incorporation of PIL tends to reduce the onset temperature of NFs decomposition, all nanofiber mats showed good thermal stability by degrading above 300 °C, allowing them to be considered as suitable materials for a wide spectrum of applications. 

**Figure 5:**
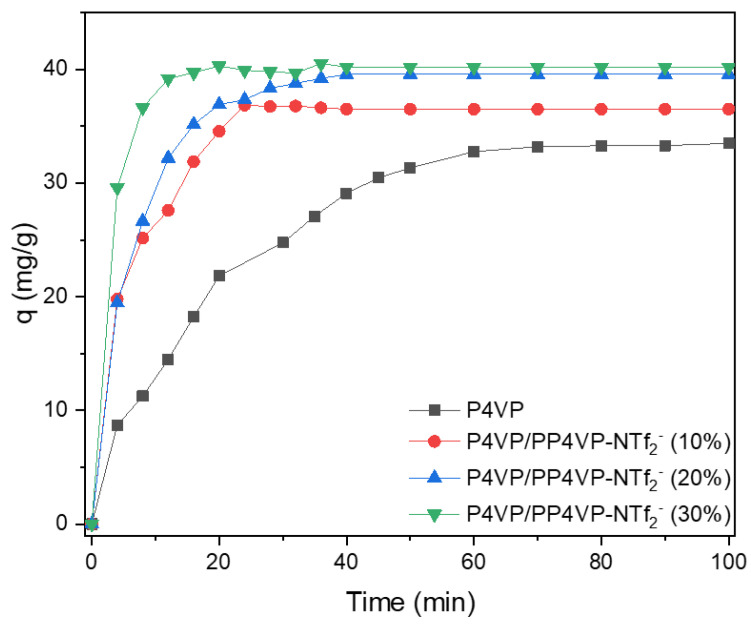
Effect of contact time on the adsorption capacity of P4VPy and P4VPy/PP4VPyNTf_2_^−^ nanofibers.

Once the characterization of the nanofibers was completed, their capacity to adsorb Au (III), in the form of the AuCl_4_^−^ anions, was studied, in order to subsequently obtain gold nanoparticles “in situ” as has been reported in previous works [26]. The kinetic of AuCl_4_^−^ adsorption onto P4VPy NFs, and NFs with different PIL contents was analyzed by plotting the adsorbed capacity (q) onto the P4VPy/PP4VPyNTf_2_^−^ nanofibers over time, Figure 5. 

From the above Figure, it can be seen that all nanofibers containing PIL showed a faster adsorption rate than P4VP NFs in the early stage of the process. In this sense, while P4VPy NFs required around 70 min to reach a plateau value, all PIL-based nanofibers achieved the above situation in remarkably less time. Additionally, an increase in the adsorption capacity was observed as the PIL content in nanofibers rises. Therefore, the inclusion of PIL proved to be an effective strategy to improve the AuCl_4_^-^ adsorption process both kinetically and in terms of capacity. 

**Figure 6:**
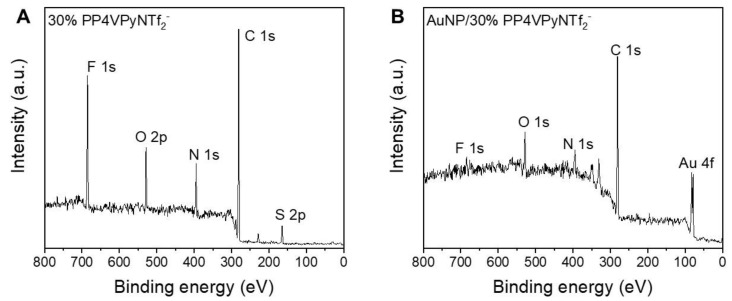
XPS survey spectra of P4VPy/PP4VPyNTf_2_^−^ nanofibers (**A**) before and (**B**) after the adsorption of gold ions.

To better understand the adsorption mechanism governing the coordination of gold ions, the experimental data shown in Figure 6 were fitted using the well-known linear expression of a pseudo-first order kinetic model (Equation (2)).

(2) $$Ln\left(q_{e}-q_{t}\right)=Ln\left(q_{e}\right)-k_{1}t$$

In this equation, *t* corresponds to the contact time between nanofibers and the AuCl_4_^−^ solution, *q_t_* and *q_e_* are the adsorption capacity at time t and at the equilibrium state (mg/g), respectively, while *k_1_* (min^−1^) is the pseudo first-order kinetic constant. The main kinetic parameters, such as *k_1_* and *q_e_*, are summarized in Table 2. 

Note that kinetic constant and q_e_ values increase as the PIL content in the nanofibrous material increases. Notably, the sample containing 30% PIL, in addition to being able to adsorb a higher amount of gold ions, exhibited a kinetic constant around six times higher than the one measured for P4VPy nanofibers. A plausible explanation to the previous results could be related to an increase in the charged species along the surface of the nanofibers containing the PP4VPyNTf_2_^−^ PIL, generating a faster adsorption of the ionic species due to electrostatic interactions. It is even reasonable to speculate the existence of an anion exchange process in which the NTf_2_^−^ entities of the PIL are replaced by AuCl_4_^−^ species as new counterions. To verify this hypothesis, XPS measurements of PP4VPyNTf_2_^−^ NFs before and after AuCl_4_^−^ adsorption were performed, and the changes in the elemental composition of the nanofiber surface were analyzed. Figure 6 shows the survey XPS spectra belonging to PP4VPyNTf_2_^−^ (30%).

The XPS spectra show the main signals corresponding to the atoms that are part of the P4VPy and PP4VPyNTf_2_^−^. As expected, the appearance of the Au 4f signal is observed after the adsorption of gold ions occurs, corresponding to the Au 4f_7/2_ and 4f_5/2_ doublet at 83.1 eV and 87.0 eV, respectively. In addition, it is interesting to note the intensity decrease ascribed to the F 1s and S 2p signals, which would evidence a substitution of the NTf_2_^−^ counterion upon adsorption of complex gold species on the surface of the fibers, suggesting an ion exchange mechanism. 

**Figure 7:**
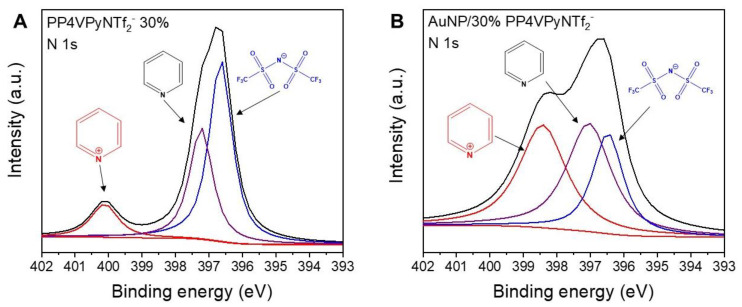
XPS deconvoluted peaks in the Nitrogen region of P4VPy/PP4VPyNTf_2_^−^ (30%) nanofibers (**A**) before and (**B**) after the adsorption of gold ions.

To achieve a deeper understanding of the adsorption mechanism, a more detailed analysis of the main atoms present in the nanofibers XPS spectrum was performed with a particular interest in the region corresponding to nitrogen 1s. This is based on reports that have previously suggested that the N atom of the pyridinic ring has significant interactions with gold atoms [27]. Figure 7 shows that the nitrogen region exhibits multiple signals that can be deconvoluted into signals with binding energies of 400.1, 397.2, and 396.6 eV, being assigned to nitrogen atoms present in quaternized pyridine rings (N^+^). In addition, it is interesting to note the intensity decrease ascribed to non-quaternized pyridine rings (N) and the counterion specie (N^−^), respectively. After gold ion adsorption, the nitrogen region undergoes remarkable changes, although the deconvolution of this region again indicated the presence of three different nitrogen species, N^+^, N, and N^−^, in this case the signals appear 398.4, 397.0, 396.5 eV, respectively. Thus, the most noticeable changes in the region of the XPS spectrum of nitrogen are, the shift of the N^+^ signal to lower binding energies, and a decrease in the intensity of the N^−^ signal. Consequently, these results would indicate the involvement of N^+^ during the adsorption process, along with the loss of some anionic species during the process. Therefore, according to the previous analysis it is possible to hypothesize that the adsorption would take place by an ionic exchange of NTf_2_^−^ by AuCl_4_^−^ species. 

Once the AuCl_4_^−^ adsorption capacity of the NFs was determined, the nanofibers containing adsorbed AuCl_4_^−^ were subjected to a reduction process for the in situ generation of gold nanoparticles. This experiment was carried out adsorbing 1% *w*/*w* of Au^+3^ considering the total mass of the nanofiber film, in order to ensure the same amount of metal in all prepared nanocomposites. 

After demonstrating the coordination capacity of these materials, the obtained nanofibers containing adsorbed AuCl_4_^−^ species were tested in the elaboration of nanocomposites by reducing the gold precursor into metal nanoparticles. The experiments consisted of the treatment of nanofiber mat samples containing 1% *w*/*w* gold ions with a sodium borohydride solution, as described in the experimental section.

**Figure 8:**
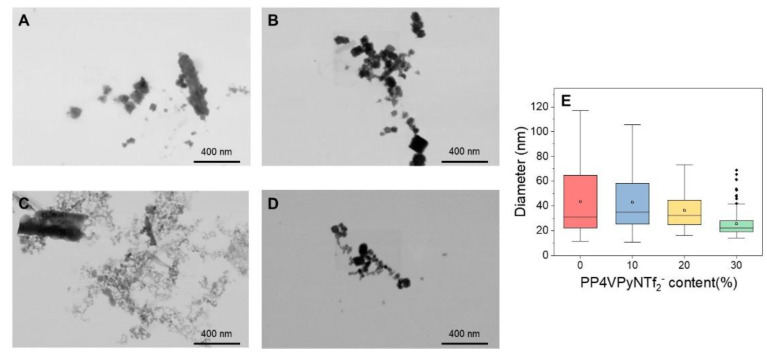
FE-SEM images of synthesized gold nanoparticles in (**A**) P4VPy, and P4VPy/PP4VPyNTf_2_^−^ with (**B**) 10%, (**C**) 20%, (**D**) 30% of PIL, and size distribution of nanoparticles diameter (**E**).

To observe the nanoparticles obtained by SEM, the organic fraction of the nanocomposites obtained was removed by calcining the nanofibers containing the nanoparticles by controlled heating up to 900 °C and depositing the residue obtained on a copper grid. The obtained images of the nanoparticles and the analysis of the corresponding size distribution are shown in Figure 8. The size of nanoparticles synthesized in situ on pure P4VPy nanofibers was approximately 43 nm with a broad size distribution, while interestingly, when the amount of PIL in NFs increases, the nanoparticle size decreases to a minimum average size of 36 nm in the case of fibers containing 30% PP4VPy-NTf_2_^−^. Notably, along with the decrease in nanoparticle size, the nanoparticles also exhibited a narrower particle size distribution as the PIL content increased. 

Finally, as detailed in experimental section, the performance of nanofibers with different PIL contents and containing gold nanoparticles as heterogeneous catalysts was evaluated. The recognized model catalytic reaction to test the catalytic activity of metal nanoparticles, reduction in 4-nitrophenol to 4-aminophenol by sodium borohydride, was used. This reduction reaction can be easily followed by UV-visible spectroscopy, due to the characteristic light absorption presented by 4-nitrophenolate species at 400 nm [28]. 

The kinetic data were determined using a pseudo-first order kinetic model, as Equation (3) indicates.

(3) $$Ln{\left[4NP\right]}_{t}=Ln{\left[4NP\right]}_{0}-k_{app}t$$

where [4*NP*]*_0_* and [4*NP*]*_t_* correspond to 4-NP concentration at the beginning of the reaction and at t minutes after this was started, respectively, *t* is the reaction time, and *k_app_* is the pseudo first-order kinetic constant of the reaction. Using the Lambert–Beer relation, Equation (3) can be converted to Equation (4).

(4) $$LnAbs_{t}=LnAbs_{0}-k_{app}t$$

where *Abs_t_* and *Abs_0_* correspond to 4-*NP* absorbance at the beginning of the reaction and at t minutes after this started, respectively. 

**Figure 9:**
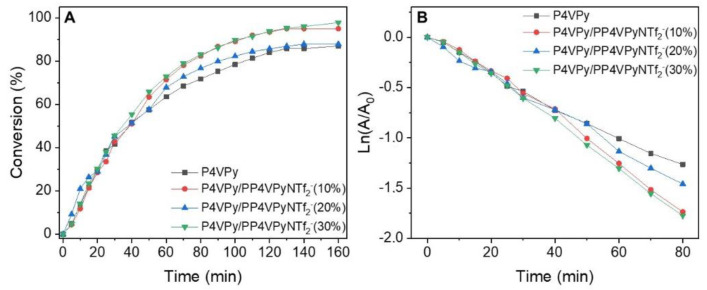

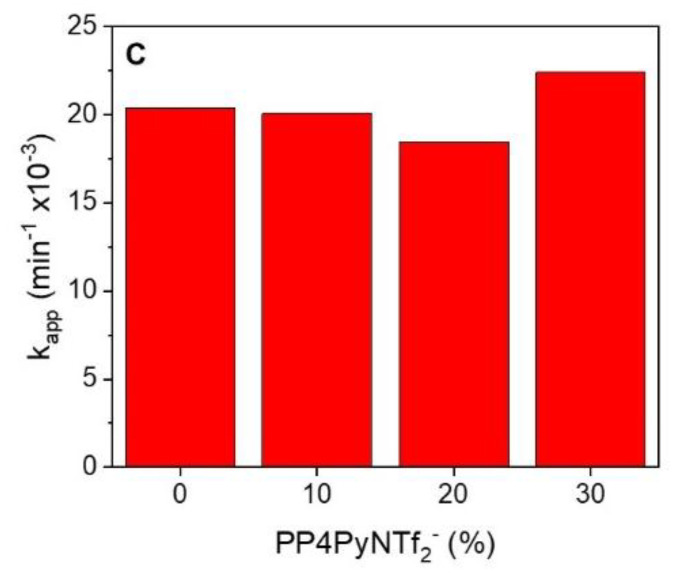
(**A**) Conversion profiles, (**B**) pseudo-first order model, and (**C**) apparent kinetic constant of the catalysts for the reduction in 4-nitrophenol into 4-aminophenol as a model reaction.

The results obtained, conversion profiles, pseudo first order models, and *k_app_* values, both for pure P4VPy nanofibers and with different poly liquid ionic contents, containing in all cases gold NPs, are shown in Figure 9.

In all cases, a rapid increase in the conversion percentage was initially observed, which slowed down as time progressed, until the highest conversion values were reached after 140 min of reaction. Interestingly, the highest value of 4 NP to 4 AP conversion (96%) was obtained with the nanofibers with the highest PIL content.

Additionally, although the values of the kinetic constants are of the same order, an evident increase in the magnitude of k_app_, corresponding to 10.5%, was observed for the reaction catalyzed with the nanofibers with 30% *w*/*w* of PIL and gold NPs, with respect to those with only P4VPy and gold NPs. 

## 4. Conclusions

PIL/PV4Py blends nanofibers with different compositions were obtained by electrospinning technique from solutions in the binary solvent DMF/DCM. Importantly, as the PIL content in the solution increased, the diameter of the NFs obtained decreased, while the amount per unit area of the same ones incremented. The process of adsorption of Au III ions and their subsequent in situ reduction, allowed to obtain Au NPs supported on PIL/P4VPy nanofibers. The presence of abundant charged groups on the polymer NFs due to the inclusion of PIL favored the adsorption process of AuCl_4_^−^, through an exchange phenomenon with the counterion NTf_2_^−^. Hybrid systems, thus obtained, (NFs-Gold NPs) showed catalytic activity in the reduction reaction of 4-nitrophenol to 4-aminophenol using sodium borohydride. In terms of catalytic activity, higher conversion values were obtained with the nanofibers containing PIL and gold nanoparticles compared to those constituted of P4VPy and gold nanoparticles. Remarkably, the highest magnitude of the kinetic constant was obtained for the nanofibers with higher PIL content.

Accordingly, the inclusion and content of PILs in NFs play a fundamental role in the catalytic efficiency and performance of the Au NPs-PIL/P4VPy NFs hybrid nanomaterial. Undoubtedly, this allows envisioning potential technological applications in the field of heterogeneous catalysis for this type of materials.

## Figures and Tables

**Table 1:** Maximum weight loss rate T_max_, onset of thermal degradation T_onset_ and percentage of final residue, of P4VP NFs, and NFs with different PIL contents.

Sample	T_max_			T_onset_	Residue (%)
	ST1	ST2	ST3		
P4VPy	392.3	/	/	382.3	5.9
P4VPy/PP4VPyNTf_2_^−^ (10%)	334.8	388.7	465.4	326.3	7.4
P4VPy/PP4VPyNTf_2_^−^ (20%)	329.0	367.7	427.3	310.6	12.3
P4VPy/PP4VPyNTf_2_^−^ (30%)	323.8	367.7	443.6	306.9	13.8

**Table 2:** Adsorption kinetics parameters for Au (III) adsorption onto nanofibers.

Sample	Pseudo-First Order		
	q_e_	k_1_ (min^−1^)	R^2^
P4VP	32.22	0.047	0.977
P4VPy/PP4VPyNTf_2_^−^ (10%)	32.58	0.049	0.973
P4VPy/PP4VPyNTf_2_^−^ (20%)	34.23	0.127	0.996
P4VPy/PP4VPyNTf_2_^−^ (30%)	37.42	0.281	0.993

## Data Availability

Not applicable.
